# A vesicular stomatitis virus glycoprotein epitope-incorporated oncolytic adenovirus overcomes CAR-dependency and shows markedly enhanced cancer cell killing and suppression of tumor growth

**DOI:** 10.18632/oncotarget.5332

**Published:** 2015-09-26

**Authors:** A-Rum Yoon, Jinwoo Hong, Chae-Ok Yun

**Affiliations:** ^1^ Department of Bioengineering, College of Engineering, Hanyang University, Seongdong-gu, Seoul 133-791, Korea

**Keywords:** oncolytic adenovirus, VSVG, fiber, CAR dependency, therapeutic efficacy

## Abstract

Utility of traditional oncolytic adenovirus (Ad) has been limited due to low expression of coxsackie and adenovirus receptor (CAR) in cancer cells which results in poor infectivity of Ads. Here with an aim of improving the efficiency of Ad's entry to the cell, we generated a novel tropism-expanded oncolytic Ad which contains the epitope of vesicular stomatitis virus glycoprotein (VSVG) at the HI-loop of Ad fiber. We generated 9 variants of oncolytic Ads with varying linkers and partial deletion to the fiber. Only one VSVG epitope-incorporated variant, RdB-1L-VSVG, which contains 1 linker and no deletion to fiber, was produced efficiently. Production of 3-dimensionaly stable fiber in RdB-1L-VSVG was confirmed by immunoblot analysis. RdB-1L-VSVG shows a remarkable improvement in cytotoxicity and total viral yield in cancer cells. RdB-1L-VSVG demonstrates enhanced cytotoxicity in cancer cells with subdued CAR-expression as it can be internalized by an alternate pathway. Competition assays with a CAR-specific antibody (Ab) or VSVG receptor, phosphatidyl serine (PS), reveals that cell internalization of RdB-1L-VSVG is mediated by both CAR and PS. Furthermore, treatment with RdB-1L-VSVG significantly enhanced anti-tumor effect *in vivo*. These studies demonstrate that the strategy to expand oncolytic Ad tropism may significantly improve therapeutic profile for cancer treatment.

## INTRODUCTION

Oncolytic adenovirus (Ad) is widely regarded as a novel and promising alternative to traditional cancer therapy, as it exhibits tumor-selective replication, high rate of viral production, and subsequently potent cytopathic effect [1–3]. Therefore oncolytic Ads are being applied in a wide range of pre-clinical and clinical trials [4, 5]. Although oncolytic Ad offers several advantages as a cancer treatment, it is limited by its native tropism which only allows cellular uptake by coxsackie and adenovirus receptor (CAR) [6, 7]. This is problematic as many of the cancer cells are CAR-deficient and thus cannot be infected by oncolytic Ad which limits the clinical efficacy of oncolytic Ad-mediated cancer gene therapy [8, 9]. Therefore, improved delivery methods are required to maximize the therapeutic potency of oncolytic Ad in cancers with subdued or ablated CAR expression.

To broaden the native tropism of oncolytic Ad, genetic modifications of Ads have been considered. Insertion of a peptide containing an arginine-glycine-aspartate (RGD) integrin recognition site to the C-terminus or HI-loop of the fiber protein markedly increases Ad infection in wide range of cells such as fibroblasts, endothelial cells, and smooth muscle cells [10]. Similarly, an addition of a cationic polylysine sequence (pK7) to the fiber knob facilitates viral binding to negatively charged cell surface molecules such as heparin sulfates and increases Ad infection to macrophages, endothelial cells, smooth muscle cells, fibroblasts, and T cells [11]. In addition to insertion of a small peptide to the fiber, other fiber modifications such as replacement of Ad5 knob to Ad35 knob, has demonstrated enhanced CD46-dependent Ad gene transfer efficiency compared to the control Ad with Ad5 knob [8, 12].

Although Ads have the potential to be genetically redirected in their tropism, most attempts to incorporate pre-identified peptide into Ad fiber have failed due to a malformation of the virus or lower affinity of binding counterpart to the surface of a target cell [13, 14]. In the three-dimensional structure, C-terminus of the fiber often points toward the virion and away from the cell surface, minimizing the efficacy of inserted targeting ligands and other motifs [15]. This structural feature of modified fiber knob also frequently causes significantly depressed fiber protein synthesis when unsuitable peptides are inserted [11, 13]. Therefore these malformed virus may aggregate in low salt concentrations which would result in aberrantly low plaque forming unit (PFU) measurements and obscure the VP/PFU ratio [16].

Furthermore, CAR binding ability can be partially impaired in fiber-modified Ad. Fiber knob is highly sensitive to deletion or addition of amino acids as deletion small as 2 amino acids drastically reduced the fiber-receptor interaction and stability [15]. Although several receptor-binding peptide motifs have been identified, the low affinity to the receptor is frequently reported which suggest that few will mediate significant increases in Ad binding to cells expressing the targeted receptor, and certain peptide motifs could prevent proper folding of the fiber protein or disrupt dimerization [17]. Therefore, it is critical to consider hindrance of targeting moiety on the three-dimensional structure of Ad fiber and identify desirable peptide with minute size for successful fiber modification of Ad to circumvent previously identified obstacles such as unstable fiber trimers and reduced cancer cell killing efficacy.

Vesicular stomatitis virus-glycoprotein (VSVG) is an envelope glycoprotein of the vesicular stomatitis virus (VSV) that enables viral entry and mediates virus attachment to the host cell. VSVG has broad cellular tropism due to its high affinity toward universally expressed surface residues which are mostly negatively charged lipids [18]. Out of these lipids, phosphatidyl-serine (PS) is broadly expressed on many cell types and has been reported to bind to the VSVG 19-mer epitope (118 to 136, GVSFNPGFPPQSCGYGSVT) which is a key negotiator for VSV's cell entry [19, 20]. Due to the wide tropism of VSVG, VSVG-pseudotyped retro- or lenti- viral vectors have been used for gene transfer to a wide variety of cells [21, 22].

Based on the biological importance of VSVG in viral entry, our previous study reported a novel Ad vector containing the VSVG 19-mer epitope at C-terminus of the fiber knob, which expanded Ad tropism and allowed viral entry via both CAR-dependent and -independent pathways. We also had shown that a VSVG epitope-incorporated Ad exhibited significant enhancement in gene transfer efficiency compared to the control Ad with a wild type fiber [23]. However, the modification of the fiber through C-terminus resulted in limited viral production with low viral titer due to highly sensitive and rigid C-terminal structure of Ad which disrupt fiber trimerization and formation of the cell-binding site localized in the knob [24]. Therefore in our current study, we hypothesized that HI-loop of the fiber knob can be employed as a convenient location for incorporation of heterologous ligands and addition of additional component such as linkers to accompany VSVG epitope which can further augment structural stability and flexibility that results in greater viral production.

Here, we constructed 9 variants of E1A- and E1B-double mutant oncolytic Ad (RdB) which incorporate VSVG-epitope with insertion of different length of linker or deletion in HI-loop of the fiber knob (RdB-VSVG). We found only one fiber-modified Ad capable of stable trimerization (RdB-1L-VSVG) with high efficiency in viral production. RdB-1L-VSVG demonstrated increased cancer cell killing efficiency, viral production, and antitumoral efficacy, in comparison to the control oncolytic Ad, RdB, in both CAR-positive and -negative cancer cells. This is the first report to demonstrate VSVG epitope-incorporated oncolytic Ad has expanded tropism that can improve Ad's infection to both CAR-positive and -negative cancer cells and strengthen its therapeutic applications.

## RESULTS

### Generation and characterization of VSVG epitope-incorporated adenovirus

Previously, we have demonstrated that the incorporation of VSVG epitope to C-terminus of Ad fiber knob allowed Ad to have a novel entry pathway into cancer cells [23]. Insertion of VSVG into C-terminus of the fiber generated a virus with highly potent antitumor efficacy and expanded tropism, yet it exhibited issues that has been previously identified with fiber modification at the C-terminus such as fiber instability and orientation of C-terminus in three-dimensional scaffold. In order to overcome certain limitations of C-terminal modifications, we have inserted the 19 aa-sequence (GVSFNPGFPPQSCGYGSVT) of VSVG epitope into the downstream of the 11^th^ threonine in the HI-loop of the Ad fiber.

**Figure 1:**
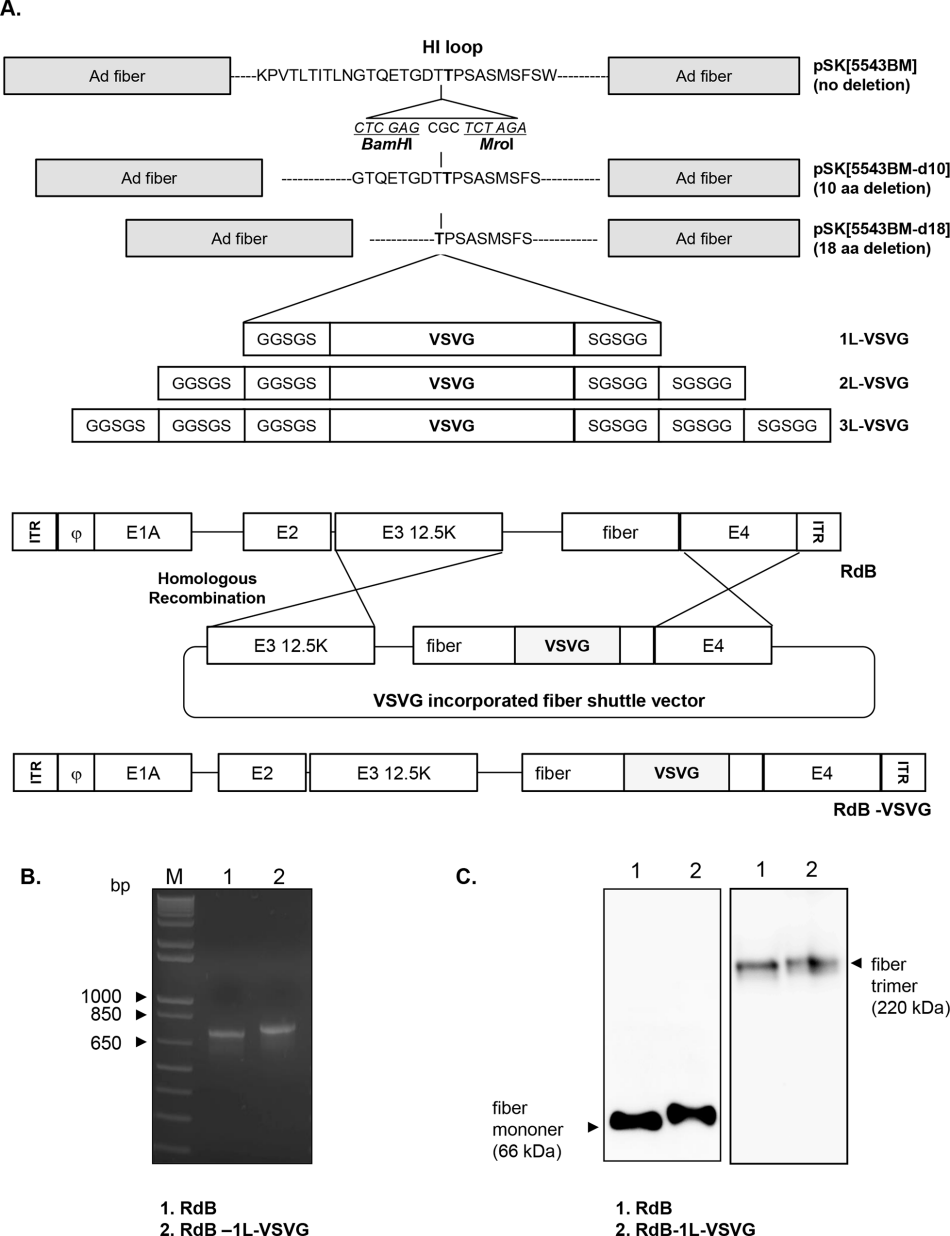
Construction of VSVG epitope-incorporated fiber-modified oncolytic Ads **A.** To construct VSVG-incorporated oncolytic Ad (RdB-VSVG), 9 variants of fiber shuttle vectors were constructed and utilized for homologous recombination with viral total oncolytic Ad vector (RdB). **B.** Polymerase chain reaction (PCR) analysis of a fiber-modified Ad (RdB-1L-VSVG). The fiber genotype was confirmed by PCR amplification with primers specific for the fiber. The 713 or 800 bp fiber genes from RdB (lane 1) or RdB-1L-VSVG (lane 2) were amplified respectively. Left lane is a DNA marker with 1-kb DNA ladder. **C.** Western blot analysis. A549 cells were infected with RdB or RdB-1L-VSVG at MOI of 10. Fiber monomer and trimer were observed under either denaturing or non-denaturing condition, respectively. Cell lysates were probed with antibodies against Ad fiber knob.

We have constructed 3 different kinds of Ad fiber shuttle vectors which have varying deletion sequence in the HI-loop (0 aa-, 10 aa-, or 18 aa-deletion), generating Ad fiber shuttle vector pSK[5543-BM], pSK[5543-BM-d10], and pSK[5543-BM-d18], respectively (Figure 1A). The fiber shuttle vector pSK5543 included intact HI-loop (KPVTLTITLNGTQETGDT**T**PSASMSFSW). pSK[5543-BM-d10] and pSK[5543-BM-d18] with 10 and 18 aa deletion in HI-loop were prepared by site-directed mutagenesis of the HI-loop. The pSK[5543-BM-d10] had GTQETGDTTPSAYSMSFS (10 aa deletion) deletion from the HI-loop prior to addition of *BamH*I and *Mro*I site. pSK[5543-BM-d18] had deletion of TPSAYSMSFS (18 aa deletion) by same method as pSK[5543-BM-dl10] and *BamH*I and *Mro*I site was inserted in place of deleted aa sequence. Further, we synthesized 3 different kinds of vectors with different copy numbers of 5 aa linker (GGSGS) (1, 2, or 3 copy) flanking VSVG epitope, generating 1L-VSVG, 2L-VSVG, and 3L-VSVG peptides, respectively. 1L-VSVG, 2L-VSVG, or 3L-VSVG were each inserted into shuttle vector pSK[5543-BM], pSK[5543-BM-d10] and pSK[5543-BM-d18], generating 9 shuttle vectors (no deletion: pSK[5543-1L-VSVG], pSK[5543-2L-VSVG], pSK[5543-3L-VSVG]; 10 deletion: pSK[5543-d10-1L-VSVG], pSK[5543-d10-2L-VSVG], pSK[5543-d10-3L-VSVG]; 18 deletion: pSK[5543-d18-1L-VSVG], pSK[5543-d18-2L-VSVG], pSK[5543-d18-3L-VSVG]). Each of the 9 different Ad fiber regions were then introduced into an E1A- and E1B-double mutant replicating Ad (RdB) genome by homologous recombination using RdB total genome plasmid and 9 different Ad fiber shuttle vectors in *E.coli*, finally constructing 9 different Ad fiber shuttle vectors in *E.coli* constructing 9 different variants of RdB-VSVG viral plasmids (Table 1).

**Table 1:** Characteristics and productions of VSVG epitope-incorporated Ads differed in the number of deleted amino acids (aa) in the HI-loop (0 aa-, 10 aa-, or 18 aa-deletion) and the number of surrounding 5 aa (GGSGS) linker sequence(s) (1, 2, or 3) on both end of the VSVG epitope

Constructed vector	Deletion in HI-loop	Number of linker (GGSGS)	Viral production
RdB	no deletion	0	Yes
RdB-1L-VSVG	no deletion	1	Yes
RdB-2L-VSVG	no deletion	2	no
RdB-3L-VSVG	no deletion	3	no
RdB-d10-1L-VSVG	10 deletion	1	no
RdB-d10-2L-VSVG	10 deletion	2	no
RdB-d10-3L-VSVG	10 deletion	3	no
RdB-d18-1L-VSVG	18 deletion	1	no
RdB-d18-2L-VSVG	18 deletion	2	no
RdB-d18-3L-VSVG	18 deletion	3	no

To generate 9 different oncolytic Ads, each of 9 different RdB-VSVG oncolytic Ad total plasmids were transfected into 293 cells. Among 9 different oncolytic Ads, only RdB-1L-VSVG was successfully generated and propagated in 293 cells. These results are in accordance with the previous studies showing that a deletion of endogenous HI-loop sequence or the addition of foreign sequence as a linker into the fiber could consequently destroy three dimensional structures of Ad fiber knob, resulting in inefficient viral production [13, 14, 25]. In order to confirm the incorporation of VSVG epitope into the HI-loop of RdB-1L-VSVG, PCR was carried out with a primer set capable of amplifying nucleotide sequence corresponding to the fiber knob. As seen in Figure 1B, a 713-bp PCR product which harbors the native fiber gene was detected in RdB PCR sample, while 800-bp PCR product was detected in RdB-1L-VSVG PCR sample. To further validate the presence of VSVG epitope in the fiber of RdB-1L-VSVG, we performed Western blot analysis using an Ad fiber-specific Ab. Under denaturing condition, fiber monomer of RdB-1L-VSVG showed higher molecular weight than that of RdB (~ 66 kDa) (Figure 1C). Further, fiber trimmers of RdB-1L-VSVG were detected as slightly bigger than those of RdB (~ 220 kDa) under non-denaturing condition, representing the incorporation of VSVG epitope into the fiber of RdB-1L-VSVG. These results suggest that the recombinant fiber of RdB-1L-VSVG can efficiently trimerize in similar manner as RdB containing wild type fiber.

### Enhanced cell killing efficiency and reduced CAR-expression dependence by RdB-1L-VSVG

**Figure 2:**
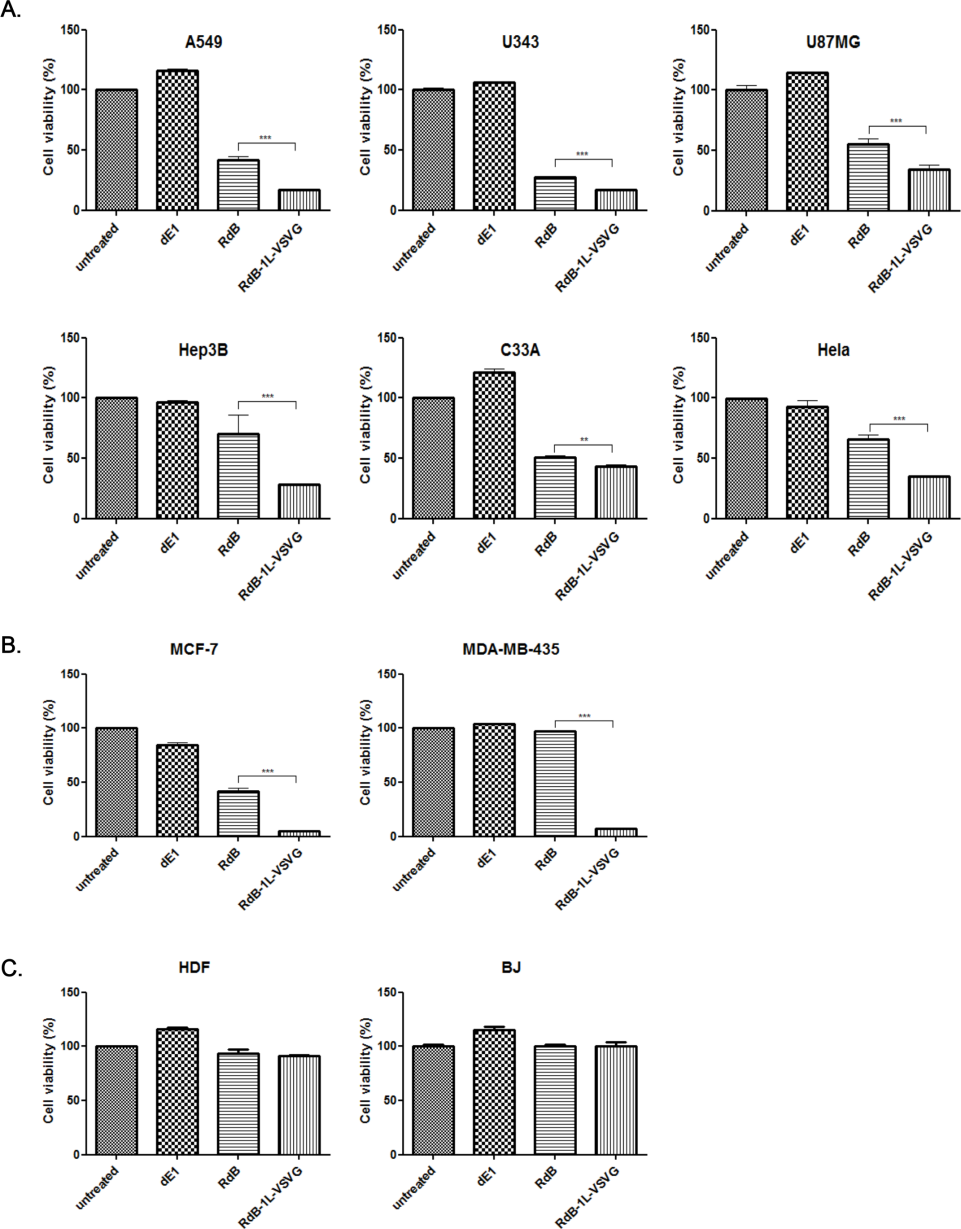
Cancer cell killing effect of RdB-1L-VSVG **A.** MTT assay in CAR-positive cancer. CAR-positive various cancer cells (A549, U343, U87MG, Hep3B, C33A, and Hela) were treated with dE1, RdB, or RdB-1L-VSVG. At 2–4 days post infection, MTT assay was performed. **B.** MTT assay in CAR-negative cancer. CAR-negative cancer cells (MCF7 and MDA-MB-435) were treated with PBS, dE1, RdB, or RdB-1L-VSVG. At 4 days post infection, MTT assay was performed. **C.** MTT assay in normal fibroblast cells. Normal fibroblast cells (HDF and BJ) were treated with PBS, dE1, RdB, or RdB-1L-VSVG. At 4 days post infection, MTT assay was performed. Each cell line was tested at least three times and data shown are representative experiments. **P < 0.01, ***P < 0.001.

To evaluate the cancer cell killing potency of the RdB-1L-VSVG, various cancer cells (A549, U343, U87MG, Hep3B, C33A, and Hela) were infected with either RdB or RdB-1L-VSVG and replication-incompetent Ad (dE1) was used as a negative control. RdB-1L-VSVG demonstrated significantly higher cancer cell killing efficacy than RdB in all tested CAR-positive cancer cells (Figure 2A). Specifically, RdB-1L-VSVG showed 59.0%, 39.2%, 38.4%, 60.0%, 15.1% or 46.4% greater cell killing potency compared to RdB in A549, U343, U87MG, Hep3B, C33A or Hela, respectively (*P* < 0.001 or *P* < 0.01). These results suggest that the insertion of VSVG motif in HI-loop of Ad fiber knob markedly enhances cancer cell killing efficacy of oncolytic Ad in CAR-positive cancer cells.

Primary cancer cells tend to express low levels of CAR and are poorly infected by Ad [8, 26]. The impact of VSVG fiber modification on CAR-independent entry mechanism was further studied using CAR-negative cancer cells (MCF7 and MDA-MB435). As shown in Figure 2B, RdB-1L-VSVG-mediated cancer cell killing efficacy was markedly enhanced compared to RdB oncolytic Ad in both CAR-negative MCF7 and MDA-MB-435 cells showing 88.8% and 92.4% greater cell killing effect, respectively (*P* < 0.001). Of note, the enhanced cancer cell killing efficacy of RdB-1L-VSVG compared to RdB was much greater in CAR-negative cells than CAR-positive cells. The cell killing ability of RdB-1L-VSVG in normal fibroblasts cells (BJ or HDF) was evaluated to confirm the cancer selectivity of RdB-1L-VSVG. As presented in Figure 2C, no apparent cell killing was observed in RdB- or RdB-1L-VSVG- infected normal fibroblasts, suggesting that the addition of VSVG epitope did not negatively affect cancer selectivity of RdB-1L-VSVG. Collectively, these results suggest that cellular receptors recognized by RdB-1L-VSVG are not limited to CAR, thus Ad vector containing VSVG epitope can provide efficient gene delivery into cells with subdued CAR expression.

### Cell entry mechanism of RdB-1L-VSVG

**Figure 3:**
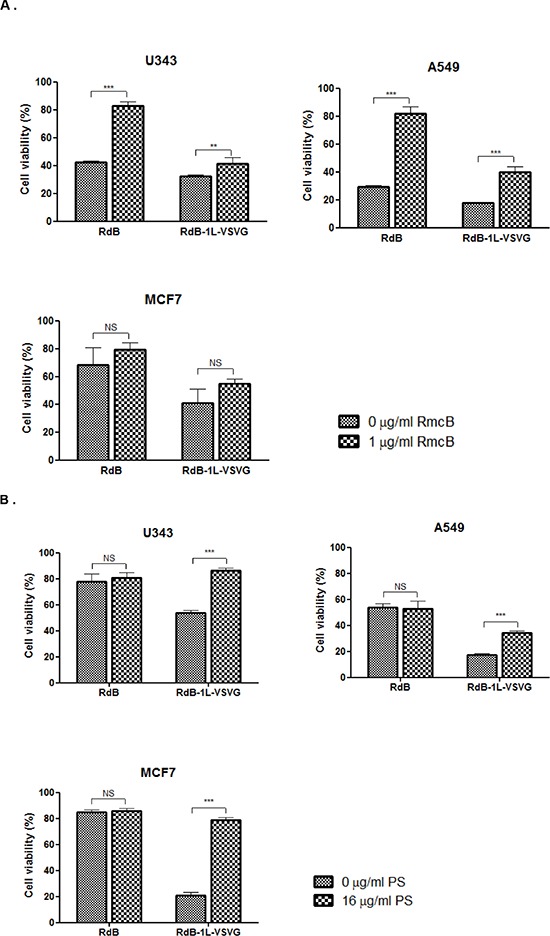
Mechanism of cellular uptake of RdB-1L-VSVG in various cancer cell lines **A.** Competition analysis with coxsackie virus and adenovirus receptor (CAR)-specific Ab. U343, A549, and MCF7 cells were pre-incubated at 4°C using CAR-specific Ab (RmcB, 1 μg/ml). RdB or RdB-1L-VSVG was then added at an MOI of 20 (for U343 and A549 cells) or 100 (for MCF7 cells), respectively. Two days later, the cell viability was determined by MTT assay. All data are presented as means ± standard deviation (SD). ***P < 0.001. **B.** Competition analysis with phosphatidyl-serine (PS). U343, A549, and MCF7 cells were pre-incubated at 4°C using phosphatidyl-serine (PS) (16 μg/ml). RdB or RdB-1L-VSVG was then added at an MOI of 20 (for U343 and A549 cells) or 100 (for MCF7 cells), respectively. Two days later, the cell viability was determined by MTT assay. All data are presented as means ± standard deviation (SD). ***P < 0.001. Each cell line was tested at least three times and data shown are representative experiments.

To further explore RdB-1L-VSVG's ability to bypass CAR-mediated pathway, we performed a competition assay with a CAR-specific Ab (RmcB). Both CAR-positive (A549 and U343) and -negative cell (MCF7) were pre-incubated with the RmcB to block the viral entry via CAR before infection with either RdB or RdB-1L-VSVG. As shown in Figure 3A, pre-treatment with 1 μg/mL of the RmcB noticeably increased cell viability by 40.5% (*P* < 0.001) in U343 cells infected with RdB compared to untreated control cells, demonstrating that CAR was efficiently blocked with 1 μg/mL of the RmcB. In contrast, RdB-1L-VSVG with pre-incubation of RmcB showed only 8.80% increase in U343 cell viability in comparison to untreated cell (*P* < 0.01), indicating that blockage of CAR-mediated entry did not significantly affect the entry of RdB-1L-VSVG. Similar results were also observed in A549 cells. However, in CAR-negative MCF7 cells, cancer cell killing efficacy was not affected by RmcB in both RdB- and RdB-1L-VSVG-infected cells due to low level of CAR-expression. In sum, these data suggest that the entry of the RdB-1L-VSVG is not solely mediated by CAR.

VSVG epitope is a key mediator of VSV entry into a broad range of host cells [19, 20]. VSVG epitope initiates cell entry of VSV by binding to PS, which is expressed on many cell types [27]. To further investigate the cell entry mechanism of RdB-1L-VSVG, we performed a competition assay with a PS in CAR-positive and -negative cells (Figure 3B). In U343 cells, there was no significant difference in cell viability between PS-treated and -untreated cells after infection with RdB. In contrast, the pretreatment of PS markedly increased viability by 32.4%, 16.7%, and 57.9% in RdB-1L-VSVG-infected U343, A549, and MCF7, respectively, compared to PS-untreated cells (*P* < 0.001), suggesting that the cellular uptake mechanism of the RdB-1L-VSVG differed from that of RdB. Of note, the enhancement of cell viability by PS was also greater in RdB-1L-VSVG-infected CAR-negative MCF cells, indicating that VSVG epitope-incorporated Ad may overcome CAR dependency by gaining an additional PS-entry capability in addition to its intact CAR-entry capability. Taken together, RdB with wild type fiber appeared to infect cells mainly by CAR-mediated entry, whereas the RdB-1L-VSVG appeared to enter cells via both CAR- and PS-mediated entry.

### Increased viral production of VSVG epitope-incorporated oncolytic Ad in cancer cells

**Figure 4:**
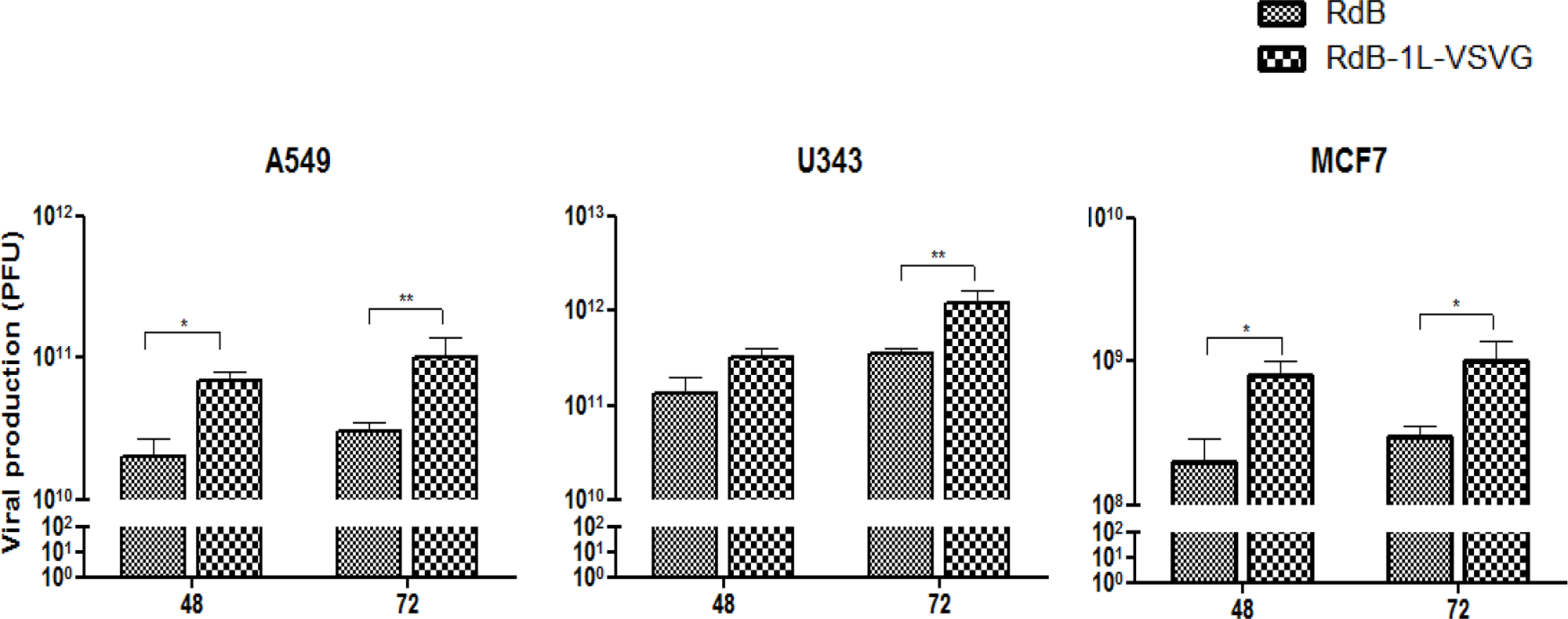
Viral production Monolayers of A549 (5 MOI), U343 (5 MOI), and MCF7 (200 MOI) cells were infected with RdB or RdB-1L-VSVG. The total virus present in cell and supernatant was extracted at 48 and 72 hours post-infection and titers were determined by TCID_50_. Viral production assay was conducted at least three times, and data shown are representative experiments.

We further explored the potential of extrication from CAR-dependent entry of oncolytic Ad to contribute for efficient viral replication. As shown in Figure 4, total viral yield of RdB and RdB-1L-VSVG was increased time-dependently, and RdB-1L-VSVG produced higher number of viral progeny than RdB. For example, RdB-1L-VSVG-infected A549 produced 4.1- and 3.5-fold higher viral progeny than RdB-infected A549 at 2- and 3-days post-infection, respectively. Importantly, RdB-1L-VSVG showed also pronounced increase in viral production compared with RdB in CAR-negative MCF7, showing 5.3- and 3.5-fold increase at 2- and 3-days post-infection, respectively. The data presented here suggest that VSVG-incorporated fiber-modified oncolytic Ad replicates efficiently and produces higher amount of progeny viruses than control oncolytic Ad with wild type fiber.

### *In vivo* therapeutic efficacy of RdB-1L-VSVG

**Figure 5:**
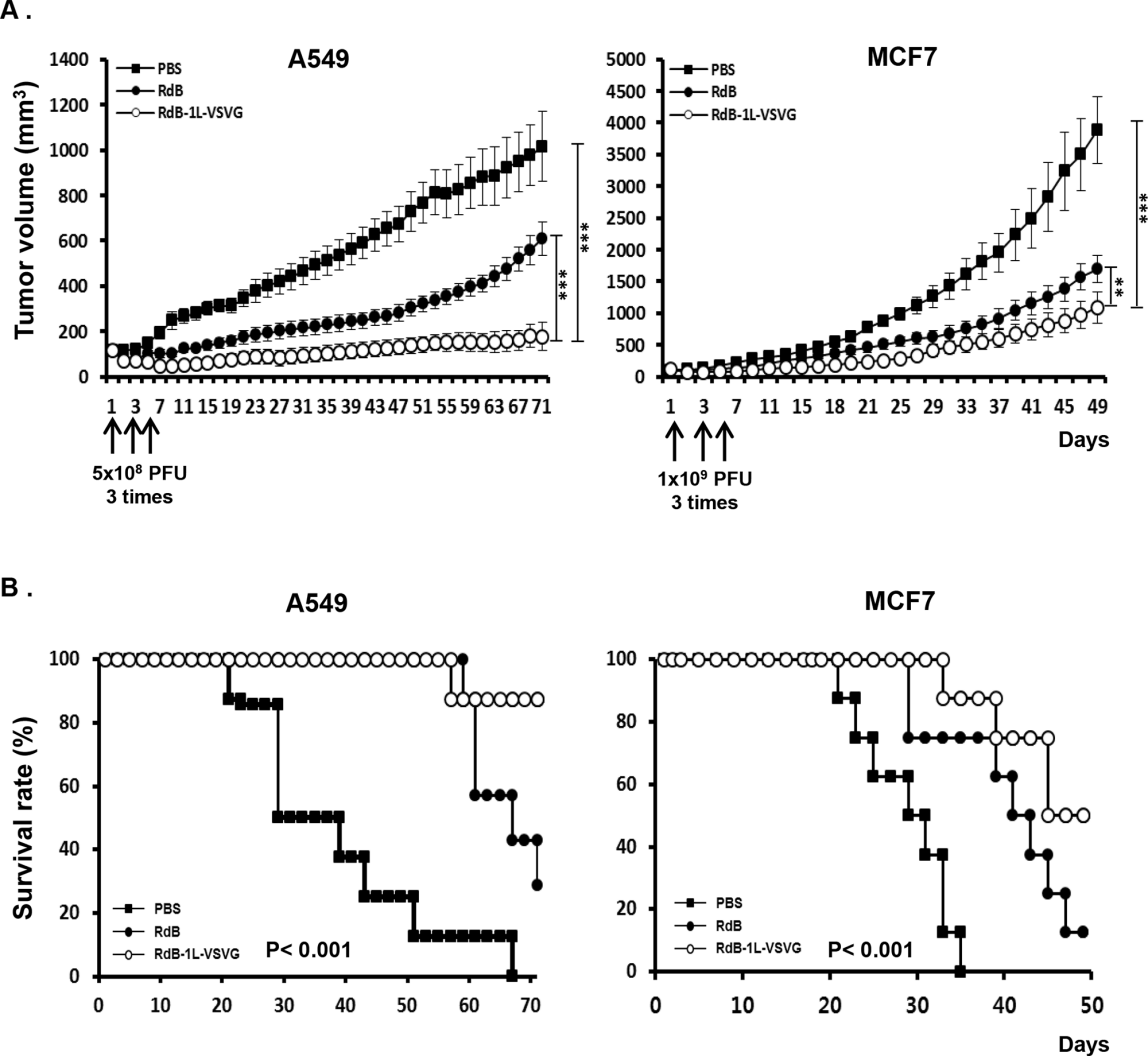
Anti-tumor effect and survival rate of oncolytic Ads in tumor xenograft model Therapeutic efficacy of RdB or RdB-1L-VSVG against A549 and MCF7 tumors established in male athymic nude mice. Tumors were established by subcutaneous implantation of 1 × 10^7^ cells and allowed to grow to an average size of 100 mm^3^. Animals with established tumors were randomized into three treatment groups of 7–8 animals each and treatment was initiated (day 1). Each group received intratumoral injection of PBS, RdB, or RdB-1L-VSVG (5 × 10^8^ PFU for A549 and 1 × 10^9^ PFU for MCF7) on days 1, 3, and 5 (vertical arrows). **A.** Evaluation of anti-tumor effect of VSVG-incorporating oncolytic Ads. Tumor growth was monitored on a 2 day interval by measuring the short and long length of the tumor. Tumor volume was estimated on the basis of the following formula: volume = 0.523 LW^2^. **P < 0.01, ***P < 0.001. **B.** Survival curve analysis of oncolytic Ads in tumor xenograft model. The percentage of surviving mice was determined by monitoring the tumor growth related events (A549; tumor size < 500 mm^3^, MCF7; tumor size < 1000 mm^3^) over a period.

We next investigated whether enhanced cancer cell killing effect of RdB-1L-VSVG translates *in vivo*. First, we assessed the relative anti-tumor efficacy of RdB-1L-VSVG in both CAR-positive (A549) and CAR-negative (MCF7) xenograft models. When the subcutaneously implanted tumors reached 80–100 mm^3^ in tumor volume, mice received intratumoral injection of PBS, RdB, or RdB-1L-VSVG at a dose of 5 × 10^8^ PFU for A549 and 1 × 10^9^ PFU for MCF7 per animal every other days for a total of three times. As seen in Figure 5A, PBS-treated A549 tumors increased up to an average size of 1015.7 ± 411.5 mm^3^ by 71 days after the treatment. In marked contrast, RdB- and RdB-1L-VSVG-treated tumors reached to an average size of 608.4 ± 160.7 mm^3^ and 177.2 ± 234.9 mm^3^ by 71 days, showing a 40.1% and 82.6% growth inhibition (*P* < 0.001), respectively. Furthermore, RdB-1L-VSVG demonstrated 70.9% better growth inhibition compared with RdB (*P* < 0.001). Likewise, by 49 days, RdB-1L-VSVG elicited more potent antitumor efficacy in MCF7 tumor model, showing 72.1% and 35.0% growth inhibition compared with PBS (*P* < 0.001) and RdB (*P* < 0.01), respectively.

Survival rate was also significantly increased in animals treated with RdB-1L-VSVG compared to RdB. By day 71 following treatment in A549 tumor-bearing mice, 87.5% of the animals in the RdB-1L-VSVG group were still viable as compared to 28.6% and 0% in the RdB- and PBS-treated group (*P* < 0.001), respectively (Figure 5B). In MCF7 tumor-bearing mice, 50% of the animals were still viable in the RdB-1L-VSVG group as compared to 12.5% and 0% in the RdB- and PBS-treated group (*P* < 0.001). Throughout the course of the study, no systemic toxicity, such as diarrhea, loss of weight, or cachexia was observed. Taken together, these all data indicate that RdB-1L-VSVG oncolytic Ad had strong inhibitory effects on tumor growth, resulting in increased survival in tumor-bearing mice.

### Enhanced apoptosis and viral replication induced by RdB-1L-VSVG

**Figure 6:**
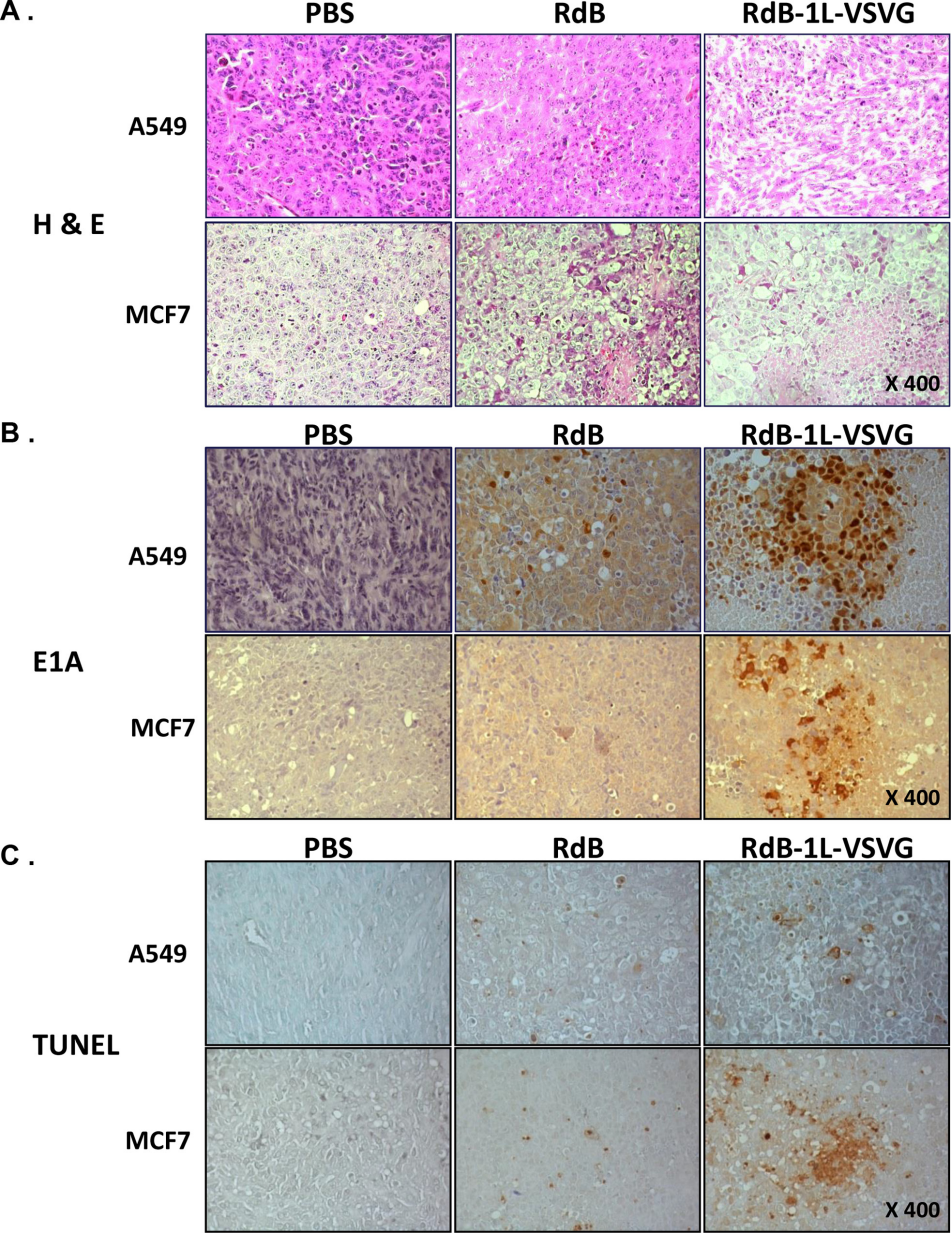

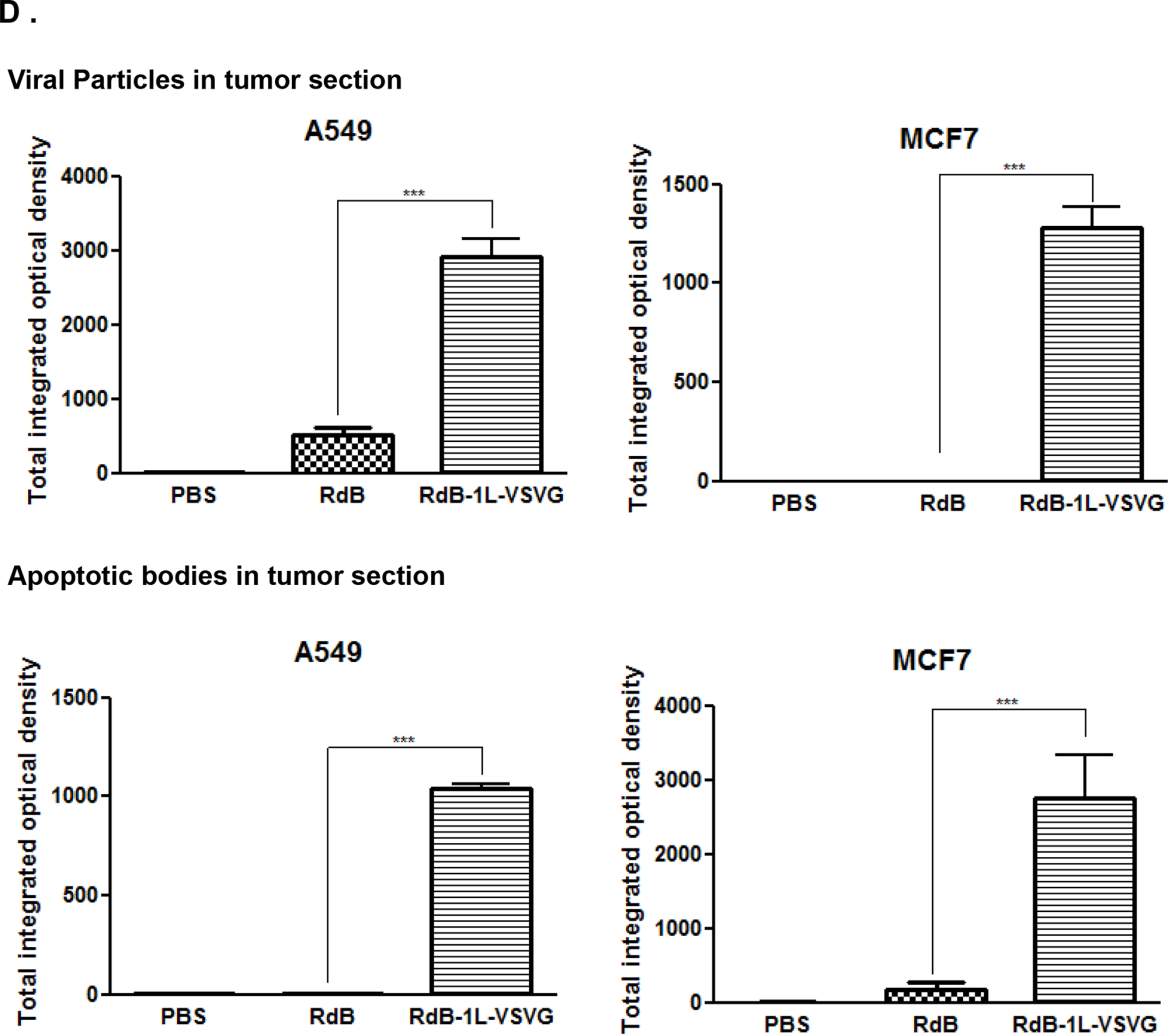
Histological analysis of tumor tissue treated with oncolytic Ads A549 and MCF7 tumors established in nude mice were injected with PBS, RdB, or RdB-1L-VSVG on day 1, 3, 5 and the tumors were harvested on day 7 for histological and immunohischemical analysis. **A.** H & E staining **B.** immunohistochemical staining of Ad E1A and **C.** TUNEL. Staining was performed in the sections of tumors treated with PBS, RdB, or RdB-1L-VSVG. **D.** Semi-quantitative analysis of E1A- or TUNNEL-stained sections using MetaMorph image analysis software. All results are shown as means ± STDEV. ***P < 0.001 compared with RdB.

The enhanced anti-tumor effect and survival benefit from VSVG-incorporated oncolytic Ad were further investigated by histological examination. Tumors were harvested from each treatment group at 3 days after the three sequential treatments. H & E staining revealed large areas of proliferating tumor cells in tumors treated with PBS (Figure 6A). Reduced numbers of proliferating tumor cells were observed and necrotic lesions were only detectable in the limited region of tumors treated with RdB. In contrast, majority of remaining tumor mass treated with RdB-1L-VSVG was necrotic. Viral replication within the tumor mass was then confirmed by immunohistochemistry using an Ab specific to Ad E1A protein. Marked increase in E1A was detected in wider areas of RdB-1L-VSVG-treated A549 tumors in comparison to RdB-treated tumors (*P* < 0.001; Figure 6B, 6D). Of particular interest was that there was significant increase in Ad E1A protein in RdB-1L-VSVG tissue whereas E1A was nearly detected in RdB-treated CAR-negative MCF7 tumor, suggesting RdB-1L-VSVG can overcome CAR-dependency for cell entry and replicates actively in CAR-negative tumor tissue.

TUNEL assay was performed to determine if active viral replication in the tumor mass promoted apoptosis in tumor tissue. Tumors treated with the RdB-1L-VSVG showed a significantly greater proportion of apoptotic body compared to that of cells in the PBS- and RdB-treated groups (*P* < 0.001; Figure 6C, 6D). These results demonstrate that RdB-1L-VSVG replicates actively in the viral injection site and spreads widely, contributing to the induction of apoptosis and necrosis. An active induction of apoptosis by RdB-1L-VSVG might also accelerate viral spread, which could ultimately lead to increased antitumor effect.

## DISCUSSION

A main obstacles of oncolytic Ad-mediated cancer gene therapy is lack of CAR expression in many types of cancer cell [8, 9], and thus oncolytic Ad vectors with expanded tropism are required. One of the strategies to overcome this challenge of oncolytic Ads is to develop vectors which can infect cancer cells on CAR-independent mode by capsid modification of oncolytic Ad [28]. The modification of capsid protein of oncolytic Ad increases expression of transgene and therapeutic efficacy in tumor cells along with active infection of oncolytic Ad [29–31]. In our current study, we modified oncolytic Ad's fiber by incorporating VSVG epitope and evaluated its potential as a cancer gene therapeutic.

Many studies have focused on developing vectors which can infect cancer cells independently of CAR by modification of the fiber [29, 31–33], capsid protein hexon [34, 35] or polypeptide IX (pIX) [30, 36]. These modifications have demonstrated enhanced transduction, infectivity, and antitumor effects compared to control Ad [8, 12]. As a viral tropism expansion strategy, we modified oncolytic Ad fiber by inserting VSVG epitope into the HI-loop. We utilized the ability of VSVG epitope, which is originally from glycoprotein G of the VSV, to increase the cell entry of oncolytic Ad through interaction with PS which is expressed on broad range of mammalian cells' surface [18, 20].

We have constructed 9 different kinds of VSVG epitope-incorporated fiber-modified oncolytic Ad viral plasmids and tried to generate oncolytic Ad from them. Out of nine oncolytic Ad variants with different length linkers and deletion of HI-loop, only one VSVG epitope-incorporated variant (RdB-1L-VSVG) with one copy of linker and no deletion in the HI-loop was successfully produced (Figure 1A, Table 1). These data indicate that the addition of VSVG epitope with one copy of linker in HI-loop of Ad fiber did not disrupt the structural formation of endogenous capsid protein, generating a functional oncolytic Ad. Consistent with previous studies showing that the deletion of endogenous HI-loop sequence or the addition of long foreign peptides into the fiber could result in poor viral production [13, 14, 25], the other eight different oncolytic Ad variants did not generate functionally active oncolyic Ads. In addition, correct formation of trimerized dodecahedra fibers are required for efficient binding to cell surface, remodeling of polarized cells, and dissemination of Ad [37]. Our results showed that recombinant fiber of RdB-1L-VSVG can efficiently trimerize (Figure 1C), thus implying that RdB-1L-VSVG with trimerized recombinant fibers can proficiently infect cells and disseminate to neighboring cells. Taken collectively, our data and previous reports demonstrate that the development of genetically targeted Ad should be performed based on a clear understanding of Ad structural biology.

In CAR-positive cancer cell lines (A549, U343, U87MG, Hep3B, C33A, and Hela) [23, 38, 39], we observed a marked enhancement in cancer cell killing by RdB-1L-VSVG compared with the unmodified oncolytic Ad, RdB. Quantitatively, RdB-1L-VSVG demonstrated a 15 to 60% enhancement in cancer cell killing in these CAR-positive cancer cell lines (Figure 2A). Of note, greater enhancements of cancer cell killing (Figure 2B; 89–92%) were detected in CAR-negative MCF7 and MDA-MB-435 cells, implying that the limitation of Ad-mediated gene transfer in CAR-negative cells can be overcome by RdB-1L-VSVG.

It is important to understand cellular uptake mechanisms of RdB-1L-VSVG for broad application of RdB-1L-VSVG in cancer gene therapy. We studied cellular uptake of the RdB-1L-VSVG by focusing on two expected main cellular uptake pathways (CAR-and PS-mediated pathways). By blocking either CAR-or PS-dependent entry of Ad, we observed that RdB with wild type fiber appeared to infect cells only by CAR-mediated entry, whereas the RdB-1L-VSVG appeared to enter cells via both CAR- and PS-mediated entry (Figure 3A, 3B), demonstrating that VSVG epitope-incorporated fiber-modified oncolytic Ad can expand tropism with high affinity of binding to PS as well as endogenous CAR. This is in a good agreement with our previous study utilizing C-terminal of Ad fiber as a epitope incorporation site, showing that dl-VSVG-LacZ's cell entry was not significantly hindered by blocking CAR [23]. The considerable evidences shown here strongly demonstrate that the addition of VSVG epitope as a receptor-binding motif to the HI-loop of Ad fiber can bypass CAR dependency of Ad via alternative cell entry mechanism.

RdB-1L-VSVG demonstrated greater cancer cell killing activity than the control RdB harboring wild-type fiber in various cancer cells (Figure 2). This is consistent with our previous results showing superior gene transgene efficiency by replication-incompetent Ad harboring VSVG epitope-incorporated fiber compared to replication-incompetent Ad harboring wild type fiber in various cancer cells [23]. Accordingly, a superior *in vivo* therapeutic efficacy as well as survival benefit was observed in mice treated with RdB-1L-VSVG compared to those treated with RdB in CAR-positive A549 tumor xenograft model (Figure 5). The enhancement of anti-tumor effect and survival rate were also replicated in CAR-negative tumor xenograft model (MCF7). As one critical point for current cancer gene therapy is high efficacy of therapeutic profile, the potent antitumor effect seen in CAR-positive and -negative tumors by RdB-1L-VSVG could make it a great candidate for future gene therapy clinical trials.

The therapeutic efficacy of oncolytic Ad critically depends on its viral replication in cancer cells. As evidenced by Figure 4, the total viral yield of RdB-1L-VSVG was 3.5- to 5.3-fold greater than that of RdB in cancer cells, suggesting that VSVG-incorporated fiber-modified oncolytic Ad infects cancer cells more efficiently than RdB which resulted in higher viral production in cancer cells. These results were also observed in tumor tissue treated in RdB-1L-VSVG, showing that RdB-1L-VSVG overcomes CAR-dependency for cell entry and exerts efficient viral replication and high progeny production in both CAR-positive and -negative tumor xenograft model (A549 and MCF7, respectively) (Figure 6B). Interestingly, it is clearly revealed that not only viral replication but also viral spreading was noticeably increased in tumor tissues treated with RdB-1L-VSVG.

TUNEL assay to evaluate the mode of cell death in tumor tissue shows that the apoptotic level was much higher in tumor tissue treated with RdB-1L-VSVG than in tissue undergoing any other treatment. We previously reported that deletion of E1B 19 kDa enhanced apoptotic effect of oncolytic Ad in tumor tissues [40]. RdB-1L-VSVG was also constructed from an Ad backbone with E1B 19 kDa-deletion of E1 gene, which contributes to its potent apoptotic activity. This apoptosis-mediated degradation of tumor mass could lead to a supportive environment with emptied spaces promoting viral spreading by the process of diffusion [41, 42]. Thus, enhanced replication profile and tissue spreading of oncolytic Ad may cause greater apoptotic activity in tumor tissue treated with RdB-1L-VSVG than RdB, which definitely leads to significantly improved antitumor efficacy in both CAR-positive and -negative tumor xenograft model (Figure 6C).

With many advantage shown in this study for usage of VSVG, we propose that VSVG epitope could alternatively be utilized in modification of Ad surface by direct conjugation with nanoparticles as a targeting ligand. Polymer-coated viral vectors have many positive aspects such as improved blood retention time and decreased trafficking to the liver [43]. One of the known obstacles is that polyplex with insufficient net cationic charge results in reduced cellular uptake as polyplex entry is heavily reliant on charge-mediated internalization due to endogenous viral receptors being masked by the polymer [43–45]. In order to overcome these important obstacles, several cell targeting moieties, such as RGD peptide, folate ligand and transferrin, have been investigated [46–48]. These targeting moieties have similar function that facilitates receptor-mediated entry of polymer complex by binding to specific cellular surface molecules. In this regards, VSVG epitope also can be an attractive candidate for conjugation with polymer which increases binding and internalization of polymers and therapeutic cargos through PS-mediated pathway.

To conclude, our study expanding targeting ability of oncolytic Ad indicate that 1) RdB-1L-VSVG can overcome the CAR dependency on their entry; 2) RdB-1L-VSVG enters to the cell via both CAR- and PS-mediated entry; 3) RdB-1L-VSVG also contribute to efficient viral replication; 4) enhanced antitumor efficacy by RdB-1L-VSVG resulted from circumvention of CAR dependency mediated by interaction between VSVG-incorporated Ad with CAR and PS expressed on the tumor cells. Overall, a strategy to combine oncolytic Ad with transductional targeting utilizing VSVG epitope has a notable therapeutic potential for targeted gene therapy.

## MATERIALS AND METHODS

### Cell lines and cell culture

Human embryonic kidney cell line expressing the adenoviral E1 region (HEK293), brain cancer cell line (U343 and U87MG), lung cancer cell line (A549), cervical cancer cell line (Hela and C33A), liver cancer cell line (Hep3B), breast cancer cell line (MCF7 and MDA-MB-435), and normal fibroblasts cells (BJ and HDF) were purchased from the American Type Culture Collection (ATCC, Manassa, VA). Both CAR-expressing cell lines (A549, U343, U87MG, Hep3B, C33A, and Hela) and CAR-deficient cell lines (MCF7, MDA-MB-435) were cultured in Dulbecco's modified eagle's medium (DMEM; Gibco BRL, Grand Island, NY) supplemented with 10% fetal bovine serum (Gibco BRL), 2 mM L-glutamine (Gibco BRL), and 100 IU/ml penicillin-streptomycin (Gibco BRL) at 37°C in a humidified atmosphere of 5% CO_2_.

### Construction of VSVG epitope-incorporated adenovirus fiber shuttle vector

To facilitate incorporation of the VSVG epitope into the HI-loop (KPVTLTITLNGTQETGDT**T**PSASMSFSW) of the fiber knob immediately downstream of 19^th^ threonine (bold), new *BamH*I and *Mro*I sites were inserted into pSK5543 [23] behind 19^th^ threonine by polymerase chain reaction (PCR)-mediated site directed mutagenesis. For PCR-mediated site directed mutagenesis, the following primer set was used: 5′-GAA ACA GGA GAC ACA *TCT AGA* GCG *CTC GAG* ACT CCA AGT GCA TAC -3′ as the sense primer and 5′-GTA TGC ACT TGG AGT *CTC GAG* CGC *TCT AGA* TGT GTC TCC TGT TTC -3′ as the antisense primer. After PCR amplification, the resulting mutated PCR product, pSK[5543-BM], containing *BamH*I and *Mro*I site (underlined) was constructed. The nucleotide sequence of mutated region was verified using an ABI PRISM 337 automatic DNA sequencer (Applied Biosystem, Foster city, CA). Two of the fiber shuttle vectors pSK[5543-BM-d18] and pSK[5543-BM-d10] were also prepared by site-directed mutagenesis of the HI-loop with 18 and 10 amino acids deletion, respectively. The pSK[5543-BM-d10] had GTQETGDTTPSAYSMSFS (10 amino acids deletion) deleted from the HI-loop prior to addition of *BamH*I and *Mro*I site. The pSK[5543-BM-d18] had TPSAYSMSFS (18 amino acids deletion) by same method as pSK[5543-BM-d10] and BamHI and MroI site was inserted in place of deleted amino acids sequence.

To construct a vector encoding 1L-VSVG, 2L-VSVG, or 3L-VSVG epitope in the HI-loop of the each fiber shuttle vector, six complementary oligonucleotides were synthesized and annealed to form a DNA duplex. Those DNA duplex were designed to contain a *BamH*I overhang 5′ end and a *Mro*I overhang 3′ end so that the fragment could be inserted into *BamH*I and *Mro*I site of pSK[5543BM], pSK[5543-BM-d10] or pSK[5543-BM-d18] vectors. The VSVG epitope consisted of 19-amino acid sequence (GVSFNPGFPPQSCGYGSVT). Sequences of the 1L-VSVG oligonucleotides are 1LF (BM); 5′-ga tcc ggc gga tct gga tcc ***gga gtt agt ttc aat cct ggg ttt cct cct caa agt tgt gga tac ggt tca gtg acg*** tcc gga agt ggt gcc t-3′ and 1LR (BM); 5′-cc gga ggc acc act tcc gga ***cgt cac tga acc gta tcc aca act ttg agg agg aaa ccc agg att gaa act aac tcc*** gga tcc aga tcc gcc g-3′. Sequences of the 2L-VSVG oligonucleotides are 2LF (BM); 5′-ga tcc ggc gga tct gga tcc ggc gga tct gga tcc ***gga gtt agt ttc aat cct ggg ttt cct cct caa agt tgt gga tac ggt tca gtg acg*** tcc gga agt ggt gcc tcc gga agt ggt t-3′ and 2LR (BM); 5′-cc gga ggc acc act tcc gga ggc acc act tcc gga ***cgt cac tga acc gta tcc aca act ttg agg agg aaa ccc agg att gaa act aac tcc*** gga tcc aga tcc gcc gga tcc aga tcc gcc g-3′. Sequences of the 3L-VSVG oligonucleotides are 3LF (BM); 5′-ga tcc ggc gga tct gga tcc ggc gga tct gga tcc ggc gga tct gga tcc ***gga gtt agt ttc aat cct ggg ttt cct cct caa agt tgt gga tac ggt tca gtg acg*** tcc gga agt ggt gcc tcc gga agt ggt gcc tcc gga agt ggt gcc t-3′ and 3LR (BM); 5′-cc gga ggc acc act tcc gga ggc acc act tcc gga ggc acc act tcc gga ***cgt cac tga acc gta tcc aca act ttg agg agg aaa ccc agg att gaa act aac tcc*** gga tcc aga tcc gcc gga tcc aga tcc gcc gga tcc aga tcc gcc g-3′. These oligonucleotide encoding each VSVG epitopes were inserted between *BamH*I and *Mro*I site of each Ad fiber shuttle vectors (pSK[5543-BM], pSK[5543-BM-d10], and pSK[5543-BM-d18]), generating 9 different of fiber shuttle vectors (no deletion: pSK[5543-1L-VSVG], pSK[5543-2L-VSVG], pSK[5543-3L-VSVG]; 10 deletion: pSK[5543-d10-1L-VSVG], pSK[5543-d10-2L-VSVG], pSK[5543-d10-3L-VSVG]; 18 deletion: pSK[5543-d18-1L-VSVG], pSK[5543-d18-2L-VSVG], pSK[5543-d18-3L-VSVG]).

### Generation of RdB-VSVG Ads

To generate the VSVG epitope-incoporated E1A- and E1B-double mutant replicating Ad, nine kinds of newly constructed Ad fiber shuttle vector pSK[5543-VSVG] series were digested with *Sac*II and *Xmn*I, and the Ad total vector RdB [49] was digested with *Spe*I for homologous DNA recombination in *E. coli* BJ5183. Proper homologous recombinant Ad DNA were digested with *Pac*I and transfected into 293 cells to generate VSVG epitope-incorporated E1A- and E1B-double mutant oncolytic Ads (no deletion: RdB-1L-VSVG, RdB-2L-VSVG, RdB-3L-VSVG; 10 deletion: RdB-d10-1L-VSVG, RdB-d10-2L-VSVG, RdB-d10-3L-VSVG; 18 deletion: RdB-d18-1L-VSVG, RdB-d18-2L-VSVG, RdB -d18-3L-VSVG). The 9 kinds of viral DNAs were transfected to generate in 293 cells and purified according to standard methods [49]. After viral generation, PCR amplification and DNA sequencing using primers specific to the VSVG epitope confirmed the genotype of the fiber. The multiplicity of infection (MOI) used in this study was determined by the absorbency of the dissociated virus at 260_nm_, where one absorbency unit is equivalent to 1.1 × 10^12^ viral particles/ml (Particles: Infectious unit (IU) ratio was 100:1). Infectious titers (plaque forming unit (PFU) per milliliter) were determined by limiting dilution assay in 293 cells [50]. The particle-to-PFU ratio for RdB or RdB-1L-VSVG were 21:1 or 24:1, respectively.

### Western analysis

To determine whether the VSVG epitope-incoporated fiber knob could form trimers, recombinant fiber was analyzed by sodium dodecyl sulfate polyacrylamide gel electrophorysis (SDS-PAGE) as previously described [23]. Briefly, cells were lysed in 50 mM Tris-HCl (pH 7.6), 1% Nonidet P-40 (NP-40), 150 mM NaCl, and 0.1 mM zinc acetate in the presence of a protease cocktail inhibitors. Proteins were loaded on 7.5% gradient gel (ELPIS-biotech, Daejeon, Korea) under either non-denaturing or denaturing condition. Fiber proteins were detected with a monoclonal Ab specific to the fiber monomer and trimer (4D2; NeoMarkers, Fremont, CA).

### MTT assay

The cytotoxicity of oncolytic Ad was determined by measuring the conversion of 3-(4,5-dimethylthiazol-2-yl)-2,5-diphenyltetrazolium bromide (MTT) to formazan. Briefly, 1–2 × 10^4^ cells were seeded into a 96 well plate at overnight and infected with RdB or RdB-1L-VSVG, along with dE1 as a negative control. After 2 to 4 days of incubation at 37°C, 50 μl of MTT (Sigma Chemical Corp, St. Louis, MO) in phosphate buffered saline (PBS) (2 mg/ml) was added to each well. After 4 hr incubation at 37°C, the supernatant was discarded and the precipitate was dissolved with 200 μl of dimethylsulfoxide (DMSO). Plates were then read on a microplate reader at 540 nm. All assays were performed in triplicate. Number of living cells was calculated from noninfected cells cultured and treated with MTT in the same condition, as were the experimental groups.

### Competition experiment

Target cells were pre-incubated for 10 min with PBS, purified CAR Ab (1 μg/ml, final concentration, in serum-free medium), or phosphatidyl serine (PS) (16 μg/ml) at 4°C before infection with either RdB or RdB-1L-VSVG (A549, U343; 20 MOI, MCF7; 100 MOI). After 20-min incubation with either RdB or RdB-VSVG at room temperature, unbound virus was rinsed off. The monolayers were then further incubated at 37°C for 2 days, and the cells were assayed by MTT assay.

### Viral production assay

The viral production of oncolytic Ad was determined by limiting titration. Cells were seeded at 3 × 10^5^ cells/well in a 6-well plate overnight, and infected with RdB or RdB-1L-VSVG at an MOI of 5 or 200; A549 and U343 at 5 MOI, MCF7 at 200 MOI. At 48 hr and 72 hr post-treatment, the media and cell pellets were subjected to three cycles of freeze/thawing in order to obtain all viruses from the infected cells and supernatant. Then total viral production was determined by TCID_50_ method on 293 cells [50].

### Antitumor effect and survivival benefit in tumor xenograft model

To assess the antitumor efficacy of oncolytic Ad, subcutaneous A549 or MCF7 tumor xenografts were established by injecting 1 × 10^7^ cells into the abdomens of 6- to 8-week-old male athymic nude mice (Orientbio, Kyunggido, Korea). Once the tumors reached 100–120 mm^3^ in volume, mice were randomized into three groups into PBS, RdB, and RdB-1L-VSVG (*n* = 7 to 8 per each group) and injected intratumoraly with PBS, RdB, or RdB-1L-VSVG (A549: 5 × 10^9^ PFU, MCF7; 1 × 10^10^ PFU) three times every other day. The length (L) and width (W) of each tumor were measured every other day with a caliper, and tumor volume was calculated according to the formula: tumor volume = 0.523 LW^2^. The percentage of surviving mice was determined by monitoring the tumor growth-related events (tumor size; A549 > 500 mm^3^, MCF7 > 1,000 mm^3^) over a period of inspection.

### Evaluation of tumor histology and immunohistochemistry

Tumor tissues were collected from mice at 3 days after the final injection, fixed in 4% formalin, and embedded in paraffin for histological and immunohistochemical examinations. Representative sections were stained with hematoxylin and eosin (H&E) and examined by light microscopy. Tumor sections were also stained with anti-rabbit Ad E1A (Santa Cruz Biotechnology, Santa Cruz, CA) to assess viral replication, respectively. After overnight incubation with primary antibodies at 4°C, tumor sections were treated with an ABC-peroxidase kit (ChemMate DAKO Envision kit; DAKO, Carpinteria, CA). All slides were counterstained with Mayer's hematoxylin. The expression levels of E1A and TUNEL-positive cells (apoptotic cells) were semi-quantitatively analyzed using MetaMorph image analysis software (Universal Image, Westchester, PA). Results were expressed as the mean optical density from 5 different digital images.

### Statistical analysis

The data were expressed as mean ± standard deviation (SD). Statistical comparison was made using two-tailed Student *t*-test, ANOVA and Kaplan-Meier method (SPSS 13.0 software; SPSS, Chicago, IL). *P < 0.05, **P < 0.01, and ***P < 0.001 were considered significant.
